# Supplementary material for: Predicting visual function by interpreting a neuronal wiring diagram
Source: Nature. 2024 Oct 2;634(8032):113–23. doi: 10.1038/s41586-024-07953-5 (PMC11446822; doi:10.1038/s41586-024-07953-5)
Supplement: Supplementary file 6 — CRF and ERF predictions for individual TmY4 and TmY9 cells. Analogous to Supplementary Data 3, but for TmY target types. Shown are the top four monosynaptic pathways, the strongest pathway passing through each of the top ten intermediary types (ranking from Extended Data Fig. 7), and the trisynaptic pathway Tm1–TmY–Dm3–TmY (see the section entitled Prediction of spatial normalization). [file 41586_2024_7953_MOESM6_ESM.zip › DataS4/TmY9q/720575940631138043.pdf]

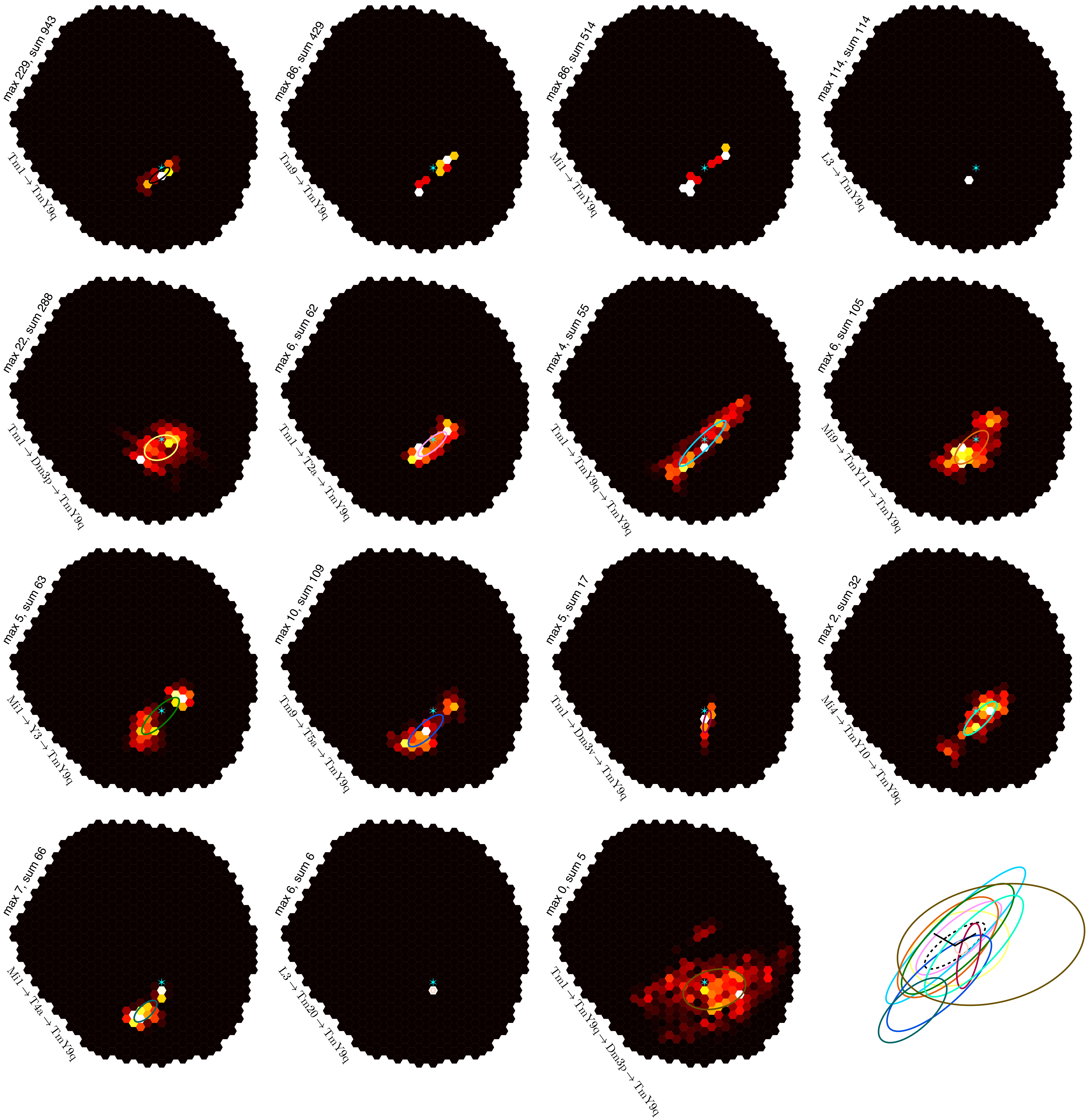
max 229, sum 943
Tm1 → TmY9q
max 86, sum 429
Tm9 → TmY9q
max 86, sum 514
Mi1 → TmY9q
max 114, sum 114
L3 → TmY9q
max 22, sum 288
Tm1 → Dm3p → TmY9q
max 6, sum 62
Tm1 → T2a → TmY9q
max 4, sum 55
Tm1 → TmY9q → TmY9q
max 6, sum 105
Mi9 → TmY11 → TmY9q
max 5, sum 63
Mi1 → Y3 → TmY9q
max 10, sum 109
Tm9 → T5a → TmY9q
max 5, sum 17
Tm1 → Dm3v → TmY9q
max 2, sum 32
Mi4 → TmY10 → TmY9q
max 7, sum 66
Mi1 → T4a → TmY9q
max 6, sum 6
L3 → Tm20 → TmY9q
max 0, sum 5
Tm1 → TmY9q → Dm3p → TmY9q
